# The Effects of Vibration and Muscle Fatigue on Trunk Sensorimotor Control in Low Back Pain Patients

**DOI:** 10.1371/journal.pone.0135838

**Published:** 2015-08-26

**Authors:** Jean-Alexandre Boucher, Jacques Abboud, François Nougarou, Martin C. Normand, Martin Descarreaux

**Affiliations:** 1 Département de Psychologie, Université du Québec à Trois-Rivières, Trois-Rivières, Québec, Canada; 2 Département d’anatomie, Université du Québec à Trois-Rivières, Trois-Rivières, Québec, Canada; 3 Département des sciences de l’activité physique, Université du Québec à Trois-Rivières, Trois-Rivières, Québec, Canada; 4 Département de chiropratique, Université du Québec à Trois-Rivières, Trois-Rivières, Québec, Canada; Universite de Nantes, FRANCE

## Abstract

**Introduction:**

Changes in sensorimotor function and increased trunk muscle fatigability have been identified in patients with chronic low back pain (cLBP). This study assessed the control of trunk force production in conditions with and without local *erector spinae* muscle vibration and evaluated the influence of muscle fatigue on trunk sensorimotor control.

**Methods:**

Twenty non-specific cLBP patients and 20 healthy participants were asked to perform submaximal isometric trunk extension torque with and without local vibration stimulation, before and after a trunk extensor muscle fatigue protocol. Constant error (CE), variable error (VE) as well as absolute error (AE) in peak torque were computed and compared across conditions. Trunk extensor muscle activation during isometric contractions and during the fatigue protocol was measured using surface electromyography (sEMG).

**Results:**

Force reproduction accuracy of the trunk was significantly lower in the patient group (CE = 9.81 ± 2.23 Nm; AE = 18.16 ± 3.97 Nm) than in healthy participants (CE = 4.44 ± 1.68 Nm; AE = 12.23 ± 2.44 Nm). Local *erector spinae* vibration induced a significant reduction in CE (4.33 ± 2.14 Nm) and AE (13.71 ± 3.45 Nm) mean scores in the patient group. Healthy participants conversely showed a significant increase in CE (8.17 ± 2.10 Nm) and AE (16.29 ± 2.82 Nm) mean scores under vibration conditions. The fatigue protocol induced *erector spinae* muscle fatigue as illustrated by a significant decrease in sEMG median time-frequency slopes. Following the fatigue protocol, patients with cLBP showed significant decrease in sEMG root mean square activity at L4-5 level and responded in similar manner with and without vibration stimulation in regard to CE mean scores.

**Conclusions:**

Patients with cLBP have a less accurate force reproduction sense than healthy participants. Local muscle vibration led to significant trunk neuromuscular control improvements in the cLBP patients before and after a muscle fatigue protocol. Muscle vibration stimulation during motor control exercises is likely to influence motor adaptation and could be considered in the treatment of cLBP. Further work is needed to clearly identify at what levels of the sensorimotor system these gains are achievable.

## Introduction

Changes in sensory function have been identified in many painful conditions of the musculoskeletal system especially among patients with chronic low back pain (cLBP) [1]. These changes, described in patients with cLBP, usually affects several physiological functions such as reduced sensory acuity [2], altered muscle recruitment patterns [3, 4] and reorganisation of the somatosensory regions of the brain cortex [5].

Local muscle vibration is often used to evaluate muscle spindle contribution to movement control. Thus, previous studies on local vibration highlight the importance of muscle spindles in proprioception [6–8]. During local vibration stimulation, a selective activation of the muscle spindles via the Ia afferent fibers is usually observed resulting in a neuromuscular response referred to the tonic vibration reflex (TVR) [9]. Some studies have suggested beneficial effects of local vibration such as significant increases in muscle activity and average mechanical power recorded during arm flexion contractions [10, 11].

Indeed, it has previously been suggested that vibration applied on the muscle belly may alter normal motor unit recruitment patterns, determining an increase in rate coding, synchronization and possibly facilitating the recruitment of faster motor units [11].

Kakigi & Shibasaki [12], in their study aiming to determine the effects of vibration on pain somatosensory evoked potentials and pain threshold, suggested that stimulation of muscle spindles under vibration influence can lead to increases in the inhibitory mechanisms of painful feeling and, therefore, be used for therapeutic purposes.

Trunk proprioception has previously been studied in individuals with low back pain using various protocols such as position sense or pointing task (motor control). Several studies have shown proprioceptive impairments in the lumbar spine of individuals reporting cLBP [1, 13, 14], but few studies have examined the local vibration effects on the performances of a trunk repositioning task in this population. In 2000, Brumagne et al. [15] suggested that when applied at segmental level L5-S1, a multifidus muscle vibration leads to a significant decrease in pelvis directional error in a sitting position as illustrated by a systematic undershooting of the target position in patients with cLBP. The same authors [16] had also concluded, in a previous study, that further research on the effect of vibration in other postures and other muscle groups was required to clarify the complex mechanism of the lumbosacral neuromuscular function. Also, a recent study conducted by Willigenburg et al. [17] found that patients with cLBP had larger errors in a spiral tracking task requiring circular trunk movements compared to healthy controls. The relative effects of lumbar muscle vibration during the motor control task in their study did not lead to any significant improvements in patients with cLBP.

Under stress conditions such as muscle fatigue or mechanical loading, lumbar proprioception may also be highly affected [14]. In a study evaluating the effect of paraspinal muscle fatigue on lumbar spine proprioception, Taimela et al. [14] found that patients with cLBP had significantly poorer ability to sense a change in lumbar position after a fatiguing protocol. It has also been shown that excessive fatigability of lumbar paraspinal muscles is a predictor of a first episode of low back pain [18] and a predictor of long-term back-related disability [19]. Indeed, it was suggested that one mechanism by which fatigue contributes to low back disorders may be spinal instability leading to injury (ie. spinal buckling) [20]. To control spinal stability, the central nervous system (CNS) must orchestrate a fine-tuned coordination of trunk muscles involving feedback (reflex) and feedforward control mechanisms [21]. Therefore, the use of vibration stimulation, known to increase spindle discharge and muscle activity, could potentially prevent decrease in muscle spindle firing rate generally observed during muscle fatigue [22] and possibly contribute to the active control of spinal stability. Although a substantial decrease in trunk proprioception under muscle fatigue condition can be expected in patients with cLBP, there is a clear lack of objective studies investigating the influence on local vibration upon back muscle fatigue and the changes in trunk proprioception.

Therefore, the primary objective of this study was to determine whether or not local vibration stimulation on *erector spinae* muscles would spontaneously yield changes in control strategy, accuracy and variability of the performance in a trunk isometric force reproduction task in patients with cLBP and healthy participants. It was hypothesized that local muscle vibration would improve trunk sensorimotor acuity in patients with cLBP and would decrease trunk sensorimotor acuity in healthy participants. The second objective of the study was to determine if vibration stimulation applied over fatigued muscles could have short-term benefits on trunk force reproduction parameters. To document the influence of muscle fatigue on trunk sensorimotor control in patients with cLBP is relevant, considering that muscle fatigue limits functionality and may hamper the involvement of cLBP patients in rehabilitation and pain management strategies [23]. The authors tested the hypothesis that muscle vibration would lead to improvements in sensorimotor control of the trunk in patients with cLBP during both the no fatigue and the post-fatigue conditions.

## Methods

### Participants

The study was conducted at the university’s neuromechanics and motor control laboratory. Sample size was estimated in order to detect a moderate effect size of 0.30 with a significance level of *P* = 0.05 and a desired power of 0.80. A minimum of 12 participants per group was needed considering the abovementioned requirements. Twenty healthy adult participants (7 women and 13 men) without any cLBP history and 20 participants (7 women and 13 men) with non-specific chronic or recurrent low back pain were therefore recruited. Patients and healthy participants were included if they were between 20 and 60 years old. Patients with non-specific cLBP were selected according to previously established criteria for chronic or recurrent low back pain (cLBP: present at least half the days over a 6-month period; recurrent low back pain: present for less than half the days over a 12-month period) [24]. Patients presenting any non-mechanical spinal condition, neurologic deficits, and chronic pain syndrome were excluded. Healthy adult participants were recruited based on the following criteria: absence of musculoskeletal or neurological symptoms related to a spine condition. Participants presenting the following conditions were also excluded: ankylosing spondylitis, trunk neuromuscular disease, inflammatory arthritis, scoliosis (15° or more) and previous spinal surgery. Before testing, each participant was informed of all experimental procedures and provided their informed written consent. All procedures were approved by the institutional Research Ethics Committee Involving Human Participants (Comité d'éthique de la recherche avec des êtres humains).

### Clinical outcomes

Clinical outcomes included the French validated version of the modified Oswestry Low Back Pain Disability Questionnaire (ODQ) to assess low back pain related disability [25] and the Tampa Scale for Kinesiophobia [26]. Clinical pain intensity of the lower back was assessed using a visual analogue scale (VAS) one week prior to testing and at the moment of testing [27].

### Preparatory procedures

**Fig 1:**
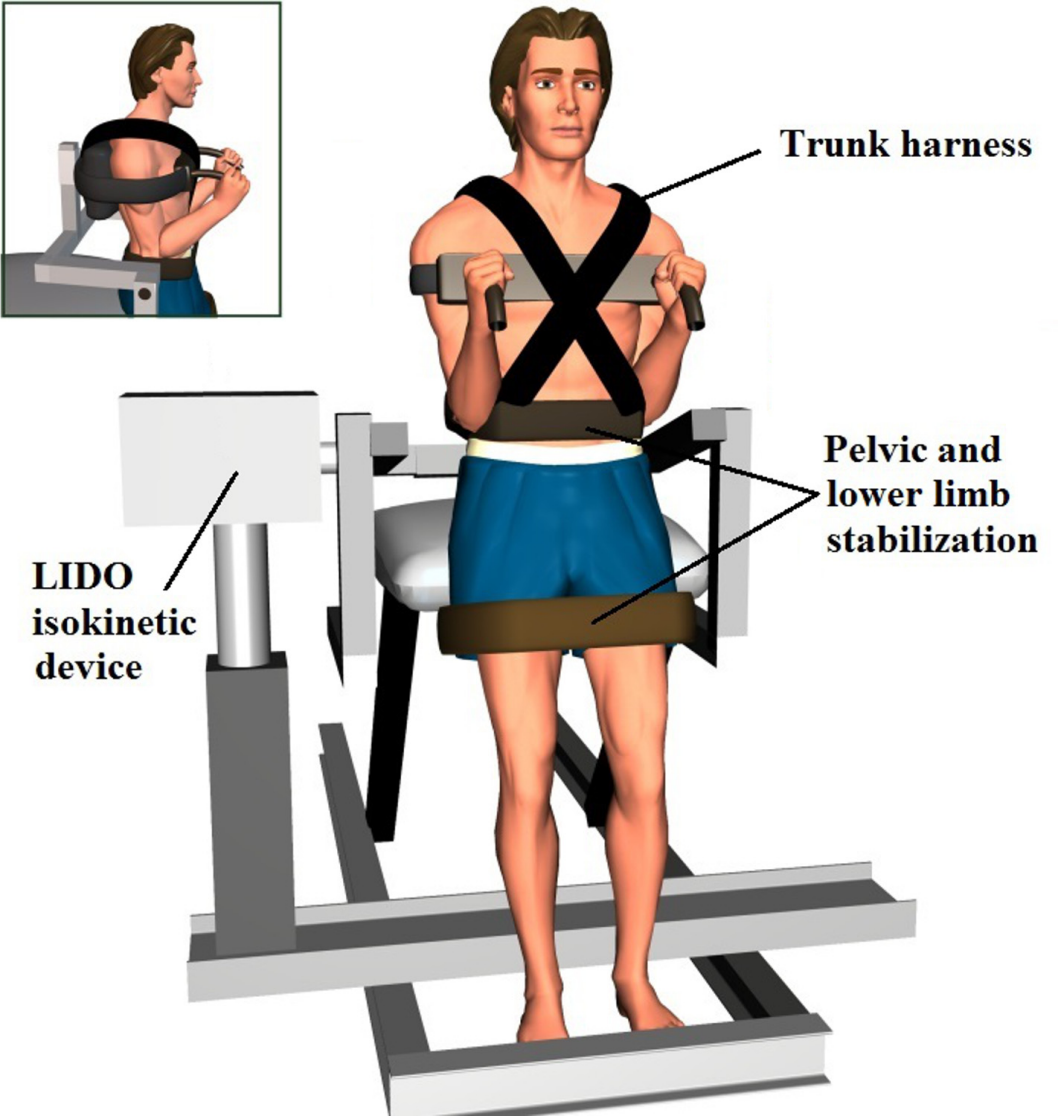
Testing position in neutral standing posture with and without *erector spinae* vibration.

Participants were tested in a neutral standing posture, without trunk flexion nor extension (Fig 1). Force data (torque) was obtained from an isokinetic device (The LIDO Active, Loredan Biomedical, West Sacramento, USA) used only in the isometric testing mode. Participants received personal encouragements from the experimenters as the maximal isometric extension torques of *erector spinae* muscle were first collected. The reference value for maximal voluntary contraction (MVC) was set at the highest torque value obtained in two consecutive 4-second trials. As a warm-up procedure, participants were then instructed to produce a sub-maximal trunk isometric force as quickly as possible. In order to ensure that all participants understood and performed the isometric force reproduction task properly, a familiarization phase was completed before the testing began, in which they were asked to produce a single impulse (shoot and release), but without attempting to correct the force once the contraction was initiated. During this phase, participants received visual accuracy feedback from an oscilloscope located in front of them, which helped them evaluate their performance to correct it for the next trial, if necessary. Participants were asked to produce peak torques set at 60% of their MVC within a 10% margin error of the target goal (ex: 100 ± 10 Nm), while keeping their eyes open for the entire session. The term peak torque, therefore, refers to the highest value of the submaximal extension torque for each trial. This familiarization phase, completed without introducing any form of vibration, was ended after participants completed ten consecutive contractions successfully. For every trial, torque data were recorded at a sampling frequency of 100 Hz. They were digitally filtered with an eighth-order Butterworth filter (10 Hz low-pass cut-off frequency).

### Muscle vibration protocol

**Fig 2:**
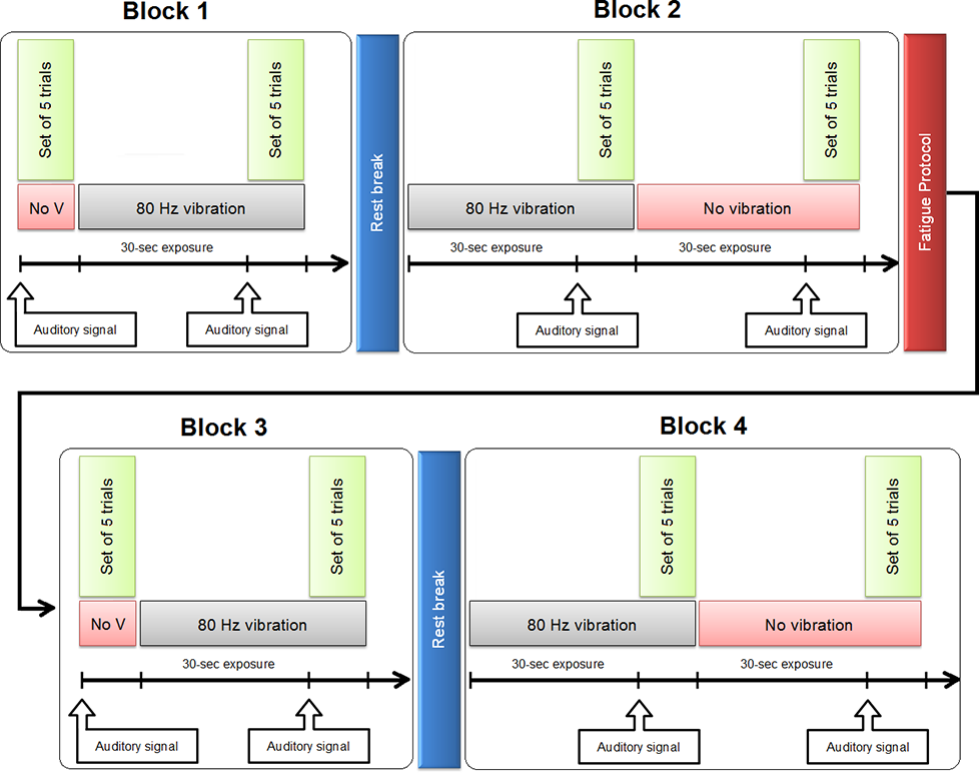
Precise timeline of the experiment. Participants were asked to perform a set of five trials (force reproduction task at 60% of their MCV) following an auditory signal which was heard every thirty seconds, for each of the vibration conditions (no vibration, 80 Hz). The order of appearance of vibration conditions (no vibration, 80 Hz vibration) differed between block 1 and block 2 (and between block 3 and block 4) to limit any vibration sequence effect.

Fig 2 represents the experiment timeline. Superficial mechanical vibration was applied perpendicularly and bilaterally on *erector spinae* muscle at the third lumbar segment level (L3) using 7.5 cm long eccentric rotating masses (4 cm diameter), weighting about 450 g. As the eccentric rotating masse rotates around the central motor shaft, the mass’ movement can be modelled as a sinusoidal wave. These vibrators, designed with a regulated DC power supply (Zurich Electric RPS-1012 MB, Taipei Hsien, Taiwan), which were held in place with a custom-made Velcro elastic lumbar belt, were placed in a standard position on all participants by the same examiner, to ensure that they were was secured with comparable tension in all tests. Vibration properties of 80 Hz with constant amplitude of 0.85 mm were the same that were used in a recent study conducted on trunk muscle [28]. Participants were asked to perform a set of five trials (force reproduction task at 60% of their MCV) following an auditory signal which was heard every thirty seconds, for each of the vibration conditions (no vibration, 80 Hz). That sequence represented one block of trials and four blocks were completed for a total of twenty trials for each vibration condition. Participants were allowed to rest for 5 minutes between each block in order to limit any fatigue effects. The order of appearance of vibration conditions (no vibration, 80 Hz vibration) differed between block 1 and block 2 (and between block 3 and block 4) to limit any vibration sequence effect. The vibration stimulation (80 Hz) was applied thirty seconds before an auditory signal was activated, and lasted through the torque generation trials, without any rest or delay. Between block 2 and 3, participants completed a trunk extensor muscle fatigue protocol corresponding to a modified version of the Biering-Sorensen test [29]. This test was performed on a 30° Roman chair, with straight upper body, the iliac crest aligned with the chair's limit, and the arms crossed on the chest in a prone position. During the fatigue task, participants were asked to hold a 11.4 kg plate to promptly induce muscular fatigue. The test was completed when the participant could no longer maintain their trunk in a straight horizontal position visually assessed (below the criterion position) or when the participant terminated the test (total exhaustion). Once the fatigue protocol completed, the participants were asked to score their perceived exertion on a 20-point Borg Scale [30].

### Electromyography

Surface electromyography (sEMG) was used to record *erector spinae* muscle activation during the isometric contractions and during the fatigue protocol. Four rigid bipolar electrodes recorded *erector spinae* muscle activation bilaterally at L4-L5 level and L1 lumbar segments. The electrodes were aligned with the muscle fiber direction and placed on the muscle belly. A reference electrode was placed on the T12 spinous process to assess internal vibration damping (essential to sEMG signals processing and noise cancellation techniques) and a ground electrode was placed over the right olecranon of all participants. Prior to application, electrical impedance of the skin at the site of electrode placement was minimized using standard skin preparation techniques. sEMG activity was recorded using a Delsys sEMG sensor sampled at 1000 Hz with a 12-bit A/D converter (PCI 6024E; National Instruments, Austin, TX).

### Data analyses

**Fig 3:**
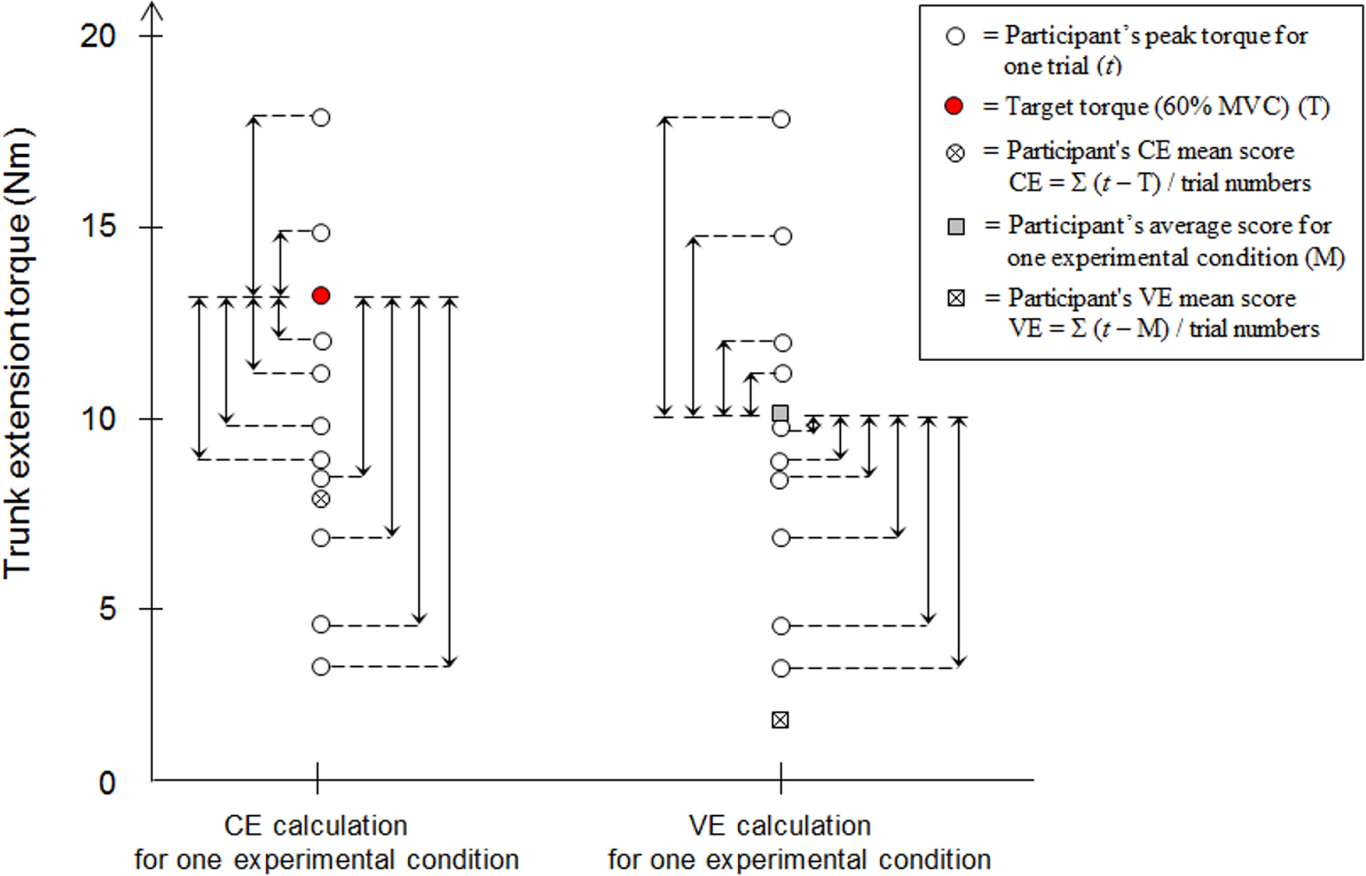
Representative data defining the Constant Error (CE) and Variable Error (VE) calculations for one experimental condition of a participant.

Time to peak torque (TPT), constant error (CE), variable error (VE) as well as absolute error (AE) in peak torque were calculated and compared between experimental conditions [31]. These variables have been successfully used in previous studies [28, 32] and they are described in more detail below. For each trial, the onset of torque and peak torque were determined, wherein onset of torque represents the initial point of the ramp up and peak torque represents the highest torque value reached for one trial. The detection and marking of these two values were made through a visual inspection by the same experimenter [32]. The TPT is defined as the time required to reach the peak torque from the onset of torque generation. The CE represents the positive or negative difference between the peak torque reached and the target torque corresponding to 60% of the MVC; it indicates the amount of direction of error relative to the target torque (measure of bias). A positive CE in trunk extension corresponds to undershooting the target torque, while a negative CE corresponds to overshooting the target torque. The VE measures the inconsistency in movement outcome and represents the variability of the participant’s performances about the mean value; it is calculated by the participant’s peak torque score on each trial and his or her own average score. Fig 3 represents an example of the CE and VE calculations for one participant. Finally, the AE in peak torque represents the average absolute deviation (without regard to torque direction) between the participant’s responses and the target torque, which accounts for both bias and variability. The variable scores (CE, VE, AE and TPT) for one participant represents his average score calculated for each experimental condition. All trials of each participant were analysed and used for the variable calculations.

sEMG data were filtered digitally by a 10- to 450 Hz band-pass, zerolag, fourth-order Butterworth filter. The data were collected by LabView (National Instruments) and processed by Matlab (MathWorks, Natick, MA). Because vibration can lead to motion artifacts resulting in spectral components at the approximate modulating frequency and its harmonics [11], the authors used a simple approach to make the sEMG root mean square (RMS) measurement insensitive to the DC power supply activation burst and vibration motion artefacts. The recorded signals for each participant’s trial were submitted to an intensive frequency domain analysis. On the basis of this analysis, vibration artefacts and related harmonics were excluded using stop-band fourth-order Butterworth filters around the modulating frequency and each harmonic.

RMS values during each trunk extension trial were calculated using time-windows corresponding to the onset and cessation of extension torque production. For each participant, mean values across conditions (10 trials) were then calculated and used to establish muscle activation patterns as suggested by previous authors [33]. RMS values were normalized with respect to the trunk extension MVC value.

To determine if *erector spinae* muscle exhibited fatigue across the modified Biering-Sorensen test, sEMG median frequency (Fast-Fourier Transform) was calculated from adjacent non-overlapping signal epochs of 1 s. The slope of the median frequency regression and its intercept were used to define the rate of change and median frequency initial values. Normalized slopes were defined as the slopes divided by the corresponding initial values and expressed in percentage per second. Localized muscle fatigue usually cause the sEMG median frequencies to decrease, yielding a negative slope [34]. The sEMG analyses were conducted by averaging normalized sEMG_RMS_ values from both sides (left and right), as used in previous studies [35, 36].

### Statistical analyses

The Shapiro-Wilk test was used to assess the distribution of the variables. All mean error in peak torque measures (CE, VE and AE) and TPT variables were normally distributed (all *P* > 0.05). This study was conducted with a counterbalanced measure design. Sampling distribution for each participant was also assessed for outlying observations (standard deviation > 3). Peak torque data and normalized sEMG_RMS_ were submitted to a 2 (groups: cLBP and control) × 2 (vibration conditions: no vibration and 80 Hz vibration) × 2 (fatigue conditions: no fatigue and post-fatigue) repeated measure analysis of variance (ANOVA), ran separately for each variable. In order to assess a potential fatigue phenomenon, pre and post-MVC values were submitted to a group × fatigue condition ANOVA with repeated measures on the last factor. Significant interactions or main effects were further analyzed using a post hoc Tukey test. sEMG median time-frequency slopes for each segmental level were submitted to a one-tailed student’s *t* Test to determine if they were significantly different than zero. The significance level was set at *P* < 0.05 for all analyses. The statistical analysis was performed with Statistica 10 (Statsoft, OK, USA).

## Results

Demographic and clinical profiles of the participants are presented in Table 1.

**Table 1:** Basic data [Mean (±SD)] on cLBP and healthy study participants.

	cLBP	Controls	*P < 0*.*05*
	*n* = 20	*n* = 20	
Age (years)	33.7 ± 14.4	29.1 ± 7.8	NS
Height (cm)	173.8 ± 11.4	174.3 ± 9.5	NS
Body mass (kg)	75.3 ± 17.5	76.4 ± 15.5	NS
ODI (%)	12.5 ± 7.8	0.0 ± 0.0	
TSK	34.3 ± 9.9	0.0 ± 0.0	
VAS pain (testing)	2.3 ± 1.8	0.0 ± 0.0	
VAS pain (1 week)	3.5 ± 1.7	0.0 ± 0.0	

*ODI* scores on the Oswestry Disability Index (maximum score = 100). *TSK* scores on the Tampa Scale for Kinesiophobia (maximum score = 68; 40/68 is considered a significant kinesiophobia). *VAS pain (testing)* pain at the moment of testing scored on the visual analogue scale (0–10). *VAS pain (1 week)* pain one week prior to testing scored on the visual analogue scale (0–10). *NS* = not significant

### Overall results

The statistical analyses yielded a significant interaction between groups, vibration conditions and fatigue conditions for the CE variable [*F*(1, 76) = 6.99, *P* < 0.01] and for the AE variable [*F*(1, 76) = 14.63, *P* < 0.001]. No significant interaction effect between groups, vibration conditions and fatigue conditions was found for the VE variable [*F*(1, 76) = 0.05, *P* > 0.05] and the TPT variable [*F*(1, 76) = 1.28, *P* > 0.05].

No significant interaction effect between groups, vibration conditions and fatigue conditions was found for the sEMG_RMS_ at the L1 level [*F*(1, 76) = 0.12, *P* > 0.05]. A vibration main effect was detected, revealing significantly higher sEMG_RMS_ activity at L1 [*F*(1, 76) = 6.81, *P* < 0.05] with the 80 Hz vibration when compared to the no vibration condition.

At the L4-5 level, no significant interaction effect between groups, vibration conditions and fatigue conditions was found for the sEMG_RMS_ [*F*(1, 76) = 0.26, *P* > 0.05]. However, a significant fatigue × vibration interaction effect [*F*(1, 76) = 9.15, *P* < 0.01], as well as a significant group × fatigue interaction effect [*F*(1, 76) = 5.81, *P* < 0.05] were found for the sEMG_RMS_. A significant vibration main effect was observed, revealing higher sEMG_RMS_ activity at L4-5 [*F*(1, 76) = 5.76, *P* < 0.05] with the 80 Hz vibration when compared to the no vibration condition. A group main effect [*F*(1, 76) = 7.23, *P* < 0.001] was also detected for the sEMG_RMS_ at the L4-5 level, showing that patients with cLBP had significantly higher sEMG_RMS_ activity than healthy controls.

### Vibration effects under the no fatigue condition

**Fig 4:**
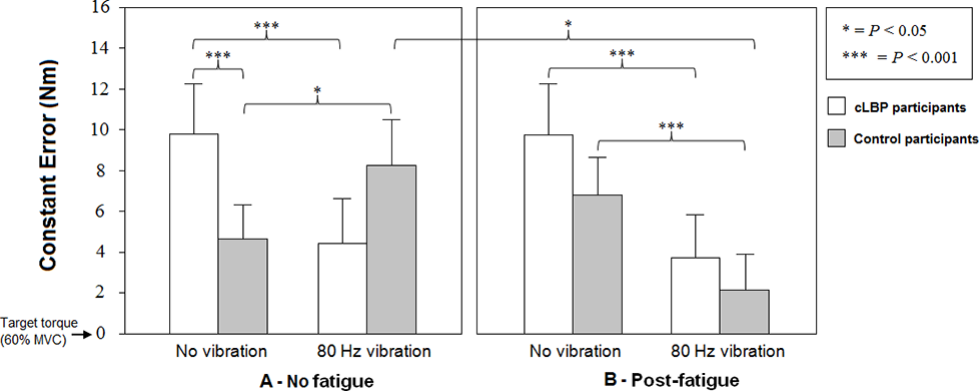
Comparison of mean constant errors for both groups in each condition (mean ± standard error)

Post hoc analyses revealed a significantly higher CE scores in patients with cLBP compared to the healthy group during the no vibration condition (*P* < 0.001). The two groups also responded differently to vibration of the *erector spinae* muscle as patients with cLBP showed a significant decrease in CE during vibration exposure (*P* < 0.001) while the control group conversely showed a significant increase in CE mean scores (*P* < 0.05) when compared to the no vibration condition (Fig 4A).

**Fig 5:**
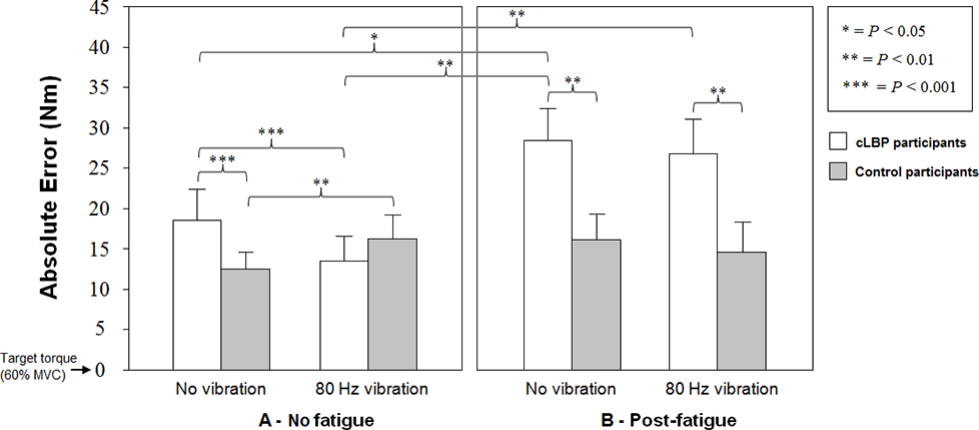
Comparison of mean absolute errors for both groups in each condition (mean ± standard error)

Post-hoc comparisons also revealed a significantly higher AE scores in patients with cLBP compared to the healthy group during the no vibration condition (*P* < 0.001). Patients with cLBP showed a significant reduction in AE under vibration exposure as compared to the no vibration condition (*P* < 0.001). In contrast, the healthy participants during the *erector spinae* muscle vibration showed significantly higher AE scores in comparison with the no vibration condition (*P* < 0.01) (Fig 5A).

**Fig 6:**
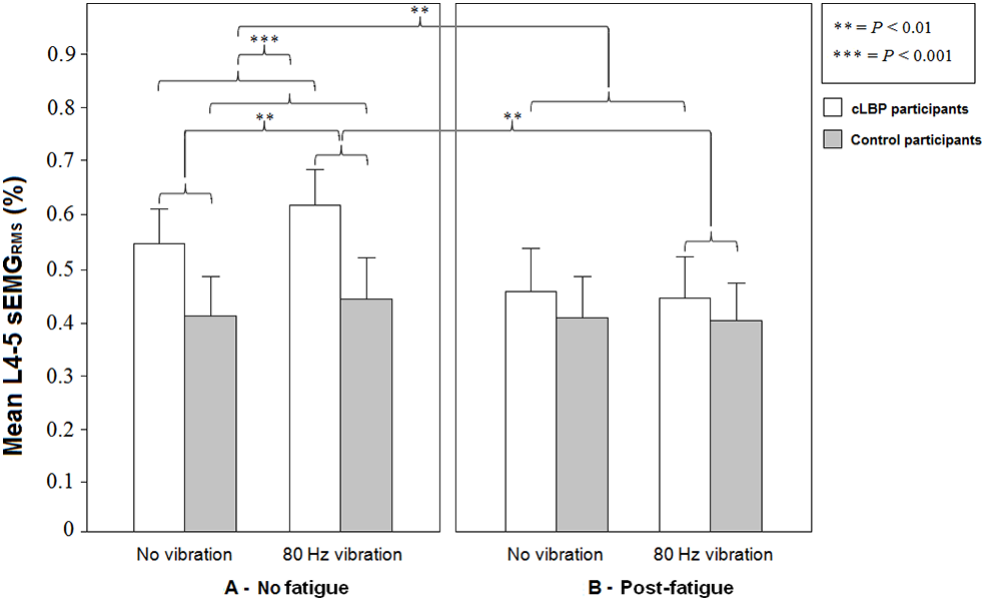
Means and standard errors of the sEMG activity of the *erector spinae* at L4-5 level for both groups in each condition. The presence of back muscle fatigue led patients with cLBP to a significant decrease in sEMG_RMS_ activity as compared to the no fatigue condition.

Post-hoc comparisons for group × fatigue interaction showed that patients with cLBP had higher sEMG_RMS_ activity at L4-5 level than healthy controls for the no fatigue condition (*P* < 0.001) (Fig 6A). Post-hoc comparisons for the fatigue × vibration interaction effect revealed a significantly higher sEMG_RMS_ activity at L4-5 level with the 80 Hz vibration when compared to the no vibration under the no fatigue condition (*P* < 0.01), independently of the group considered (Fig 6A).

### Fatigue effects

The presence of back muscle fatigue led patients with cLBP to a significant decrease in sEMG_RMS_ activity at L4-5 level as compared to the no fatigue condition (*P* < 0.01), independently of the vibration condition (Fig 6).

Statistical analysis yielded a main effect of fatigue for pre and post-MVC values (*P* < 0.01). The average MVC (± SD) in trunk extension for patients with cLBP was 137.53 ± 73.24 Nm before the fatigue protocol and 123.34 ± 68.44 Nm immediately after the fatigue protocol, indicating a 10.32% decrease in MVC (*P* < 0.05). The average MVC in trunk extension for healthy participants was 130.79 ± 56.68 Nm before the fatigue protocol and 109.82 ± 48.44 Nm immediately after the fatigue protocol, indicating a 16.03% decrease in MVC (*P* < 0.05). No significant difference between groups was observed for MVCs before and after the fatigue protocol (all *P* > 0.05).

Important fatigue-related changes were observed during the Biering-Sorensen protocol. sEMG time-frequency analysis at the L1 and L4-5 paravertebral electrode sites for both groups indicated that linear regression slopes were all negatives (Table 2). Student’s *t* Test revealed no significant difference between both groups for the total holding time, and for the L1 and L4-5 linear equation slopes (all *P* > 0.05). One-tailed *t* Tests revealed significant muscle fatigue at each segmental level for both groups (all *P* < 0.05). Table 3 presents the participant’s perceived exertion during the fatigue protocol.

**Table 2:** Mean (±SD) sEMG median time-frequency slope at L1 and L4-5 levels and modified Sorensen total holding time in sec for both groups.

	cLBP	Controls	*P < 0*.*05*
	*n* = 20	*n* = 20	
L1 segmental level	-0.16 ± 0.09	-0.17 ± 0.07	NS
L4-5 segmental level	-0.35 ± 0.15	-0.32 ± 0.15	NS
Total holding time (sec)	118.6 ± 44.1	123.6 ± 28.5	NS

**Table 3:** Mean (±SD) level of perceived exertion during the fatigue protocol.

	cLBP	Controls	*P < 0*.*05*
	*n* = 20	*n* = 20	
**The Borg Scale**	16.4 ± 2.1	16.9 ± 1.8	NS

*The Borg Scale* starts with “no feeling of exertion”, which rates a 6, and ends with “very, very hard,” which rates a 20. Moderate activities register 11 to 14 on the Borg scale (“fairly light” to “somewhat hard”), while vigorous activities usually rate a 15 or higher (“hard” to “very, very hard”). *NS* = not significant

### Vibration effects under the fatigue condition

Following the fatigue protocol, healthy participants showed a significant decrease in CE during muscle vibration when compared to the no vibration condition (*P* < 0.001). A decrease in CE scores during vibration was also observed in patients with cLBP (*P* < 0.001) (Fig 4B). During vibration exposure, a difference was observed between the no- and the post-fatigue condition for the control group (*P* < 0.05).

There were significant differences for both the vibration and no vibration conditions where cLBP patients showed significantly higher AE scores as compared to the healthy participants (all *P* < 0.01). Following the fatigue protocol, AE scores in patients with cLBP were significantly higher than the no fatigue scores for the vibration (*P* < 0.01) and the no vibration conditions (*P* < 0.05) (Fig 5B).

The presence of back muscle fatigue led to a significant decrease in sEMG_RMS_ activity at L4-5 level for the 80 Hz vibration condition, independently of the group considered (Fig 6). Finally, a main effect of fatigue was found for the TPT variable [*F*(1, 76) = 16.76, *P* < 0.001] where values for both group were significantly higher in the post-fatigue condition regardless of the vibration conditions. Participants from both groups took more time, therefore, to reach the peak torque following the fatigue protocol.

## Discussion

The aim of the present study was to assess the performance accuracy and variability of trunk reproduction force in conditions with and without *erector spinae* muscle vibration, and to evaluate the influence of muscle fatigue on trunk sensorimotor control in patient with cLBP and healthy participants.

The present study included a group of cLBP patients with an average mild pain level score (2.3 ± 1.8) at the moment of testing [37, 38]. Results showed that patients with cLBP had significantly lower trunk isometric force reproduction accuracy than the healthy participants. Higher CE and AE mean scores found in patients with cLBP clearly support this observation. Similar findings have been previously reported by Brumagne et al. [15], who found that patients with cLBP had a less refined lumbosacral position sense than healthy individuals in a sitting position. The results reported by Brumagne et al. [15] provide evidence for reduced trunk neuromuscular control during dynamic contractions in patients with cLBP, and the results of the present study provide evidence for reduced trunk neuromuscular control during isometric contractions in patients with cLBP. It is worth mentioning that measures of error such as CE, AE and VE mean scores reported in the present study and the one conducted by Brumagne et al. [15] are considered as outcome measures and not process measures (see Schmidt & Wrisberg [39] for detailed description).

Sensorimotor disturbances of the spine could result from modifications in somatosensory afferent activity, which can be due to trauma or to the modulatory effect of pain and sympathetic activation on muscle spindle sensitivity [40]. Consistent with this explanation, Myers et al. [41] suggested that increased afferent signals sent by pain receptors are believed to override and subsequently decrease proprioception afferents. Reweighting of sensory signals based on location have also been demonstrated in patients with cLBP as they seem to adopt a body and trunk stiffening strategy and rely more on lower limb proprioception [42, 43]. In the present study, it is therefore possible that patients with cLBP, having limited somatosensory information from back muscle, had to reweight sensory information from other segments or muscle groups. This way, distorted afferents from back muscle could have been compensated by other undistorted afferents originating from the pelvic girdle and lower limb muscles leading to lower trunk isometric force reproduction accuracy.

### Vibration effects

Vibration of the *erector spinae* muscle induced a significant reduction of the CE and AE mean scores in patients with cLBP. The accuracy with which patients with cLBP reproduced a trunk sub-maximal force was, therefore, improved during vibration stimulation when compared to the no vibration condition. This acute effect of local muscle vibration in patients with cLBP has several possible explanations. Hollins et al. [44], in their study on the ability of vibration to compromise detection of a nociceptive stimulus, found that vibrations ranging from 20 to 230 Hz were able to modulate nociception by reducing the noxious stimulus sensitivity. They also concluded that no mechanoreceptive channel appears to have a privileged role in antinociception. Some authors also suggested that muscle vibration could distort muscle’s primary afferent by introducing a bias signal in a parallel channel, because vibration can modestly active the Pacinian corpuscle and others cutaneous mechanoreceptors [7, 45, 46]. Although the neurophysiological mechanism for such changes remains unclear, it is possible that vibration stimulation in this study may have improved the muscle spindle function in patients with cLBP therefore improving somatosensory information processing. By stimulating Ia afferents and modulating the nociceptive pathways activation, vibration stimulation may lead to a transient sensory reweighting of the *erector spinae* muscle resulting in significant improvements in trunk neuromuscular control.

It is well known that mechanical vibration administered to tendons or muscles can induce a reflex response [9]. The study conducted by Nakajima et al. [47] suggests that 80 Hz vibration during muscle contraction can lead to specific TVR increases observed with higher sEMG_RMS_ activity of the biceps. In the present study, local vibration application led to significant increase in *erector spinae* muscle activity at both L1 and L4-5 segmental levels. These results could partly be explained through the TVR effect. Regardless of the experimental conditions, patients with cLBP in this study also showed higher muscle activity than healthy controls at L4-5 level but not at L1 level. This is consistent with the ‘‘redistribution of activity within muscle” theory that has been described in patients with cLBP [48]. It is difficult to explain the improvements in force reproduction accuracy of the trunk observed in patients with cLBP by the vibration-related increase in muscle activity, since healthy controls also showed significant increases in muscle activity during vibration but with higher CE and AE mean scores. It is suggested, therefore, that clinical efficacy of local vibration during a trunk sensorimotor task in patients with cLBP is not directly linked to the *erector spinae* muscle sEMG activity. It is also possible that the pain alleviation usually reported during vibration might have led to a significant reduction in the fear-avoidance behavior during the force reproduction task [49].

Conversely, vibration stimulation significantly decreased the healthy participants’ accuracy during the force reproduction task. This finding is in accordance with a previous study conducted on healthy participants in which *erector spinae* muscle vibration interfered with torque generation sequence by distorting proprioceptive information resulting in muscle lengthening illusion [28].

### Muscle fatigue and vibration

Previous studies have shown that back muscle fatigue is usually accompanied by a diminished control of trunk movements [50] and an altered coordination of trunk muscle activities [51], as well as reductions in accuracy when trying to generate a given force [52]. It is also well established that patients with cLPB have excessive fatigability of the back muscles which is probably a consequence of pain, rather than a cause [53, 54]. From a theoretical point of view, these deficits, in association with cLBP, may leave the lumbar spine more susceptible to reinjury. Therefore, the purpose of creating a fatigue condition in the present study was to determine if vibration stimulation applied over fatigued muscles could have short-term benefits on trunk force production parameters. The results of the present study suggest that the back extensors fatigue protocol clearly induced muscle fatigue for both groups. However, the level of muscle fatigue between cLBP and healthy participants did not differ. Under the influence of vibration, patients with cLBP showed significant reduction of the CE values, which suggests accuracy improvements in the force reproduction task. AE values with and without vibration stimulation, however, were higher during the post-fatigue condition. A controversy exists, however, about the use and interpretation of AE. The mathematical properties of AE have been shown to be a complex combination of CE and VE, and it remains difficult to precisely assess the relative contribution of each measurement to AE [31]. Based on CE mean score, these findings suggest that acute *erector spinae* vibration may enhance sensorimotor acuity in patients with cLBP in conditions with and without back muscle fatigue, and could, therefore, be considered as an adjunct to actual rehabilitation strategies. It is suggested that vibration stimulation during rehabilitation exercises could potentially reduce the risks related to back muscle fatigue and participate in preventing lumbar spine reinjury.

Interestingly, healthy participants during the post-fatigue condition and under vibration stimulation showed significant decreases in CE when compared to the no fatigue condition. Theoretically, muscle spindle discharge decreases during fatiguing static contraction [22]. Consistent with this change, motor units usually demonstrated a decrease in firing rate during muscle fatigue [55]. Griffin et al. [56], in their study on vibration and motor unit firing rate, found that application of periodic muscle vibration maintained the motor unit firing rate during submaximal fatiguing isometric contractions. These authors also concluded that muscle vibration can enhance the excitatory input from Ia afferents to the motoneuron pool and transiently restore the motor unit discharge rate. In the present study, the significant decrease in sEMG activity at L4-5 level observed following the fatigue protocol could not, however, be prevented by local vibration stimulation. Although the hypothesis that fatigue-related modulation of motor unit firing rate during submaximal contractions could be prevented by vibration in healthy controls received initial support [56], the present findings do not provide any evidence to confirm this assumption. Even though it is not of clinical importance, alternative theories are required to explain the improvement in sensorimotor control under vibration stimulation following a fatigue protocol in healthy controls.

Higher TPT scores were found in all post-fatigue conditions for both groups, suggesting a modified control strategy developed under exposure to muscle fatigue. This result may be closely related to the neuromuscular effects of fatigue on muscle activation, including reduction in the ability of muscle to produce force and power.

### Clinical considerations

A spectrum of clinical interventions has been proposed to retrain motor control in the presence of musculoskeletal pain and disability. The present findings suggest that 80 Hz vibration stimulation in patients with cLBP is likely to influence motor adaptation of the sensorimotor system, leading to improved accuracy in isometric force production parameters. Bosco, Cardinale [10], in their study on local vibration on mechanical power and sEMG activity, concluded that vibration stimulation was able to stimulate the neuromuscular system more than others treatments used to improve neuromuscular properties. *Erector spinae* muscle vibration could, therefore, be used as an additional stimulation to the sensorimotor system during rehabilitation exercises. The sensory integration process may increase the contribution on *erector spinae* sensory afferents during motor control exercises performed under vibration stimulation (sensory reweighting). The results of the present study also showed that vibration effects on trunk sensorimotor control (increases in force production accuracy) may operate similarly with and without back extensors muscle fatigue. These findings suggest that vibration may lead to beneficial effects at multiple stages of the rehabilitation process where patients with cLBP present different levels of muscle fatigue. Even if *erector spinae* vibration stimulation suggests short-term benefits on neuromuscular control, potential long-term benefits involving primary outcomes such as spine loading, movement and motor variability need to be further investigated.

### Limitations

A potential limitation of the present study is that results may suggest rapid but transient adaptations to muscle vibration, resulting in changes in isometric force production parameters. Although such adaptation is mainly driven by changes in muscle spindle discharge, changes in sensorimotor integration or motor planning remain to be determined. The experimental design of this study, however, cannot provide specific information with regard to the contribution of central pathways and mechanisms aiming at identifying the complete proper neural mechanisms. A second limitation is the sEMG frequency components and the harmonics related to vibration that were excluded from the sEMG_RMS_ calculation. Excluding vibration-related frequencies may have removed physiological signals from the muscle. Further research should focus on the optimal dose relationship of vibration duration on neuromuscular and performance aspects for cLBP populations. In addition, more studies are needed to determine whether these responses correlate with long-term clinical outcomes.

## Conclusion

The findings of this study suggest that patients with cLBP have a less accurate force generation sense than healthy individuals, presumably because of altered *erector spinae* muscle spindle afferents. Local muscle vibration led to significant trunk neuromuscular control improvements in the cLBP patients, which suggests that the weighting of proprioceptive feedback from *erector spinae* muscle spindles differs between groups. The significant increase in neuromuscular control observed for the patients in the non-fatigued condition supports the clinical evidence of improved trunk function during vibration application, and contributes to the efficacy of this approach in the management of patients with cLBP. Local muscle vibration also led to significant trunk neuromuscular control improvements in the cLBP patients following a muscle fatigue protocol.
